# Identification of predictors for neurological outcome after cardiac arrest in peripheral blood mononuclear cells through integrated bioinformatics analysis and machine learning

**DOI:** 10.1007/s10142-023-01016-0

**Published:** 2023-03-17

**Authors:** Zhonghao Li, Ying Qin, Xiaoyu Liu, Jie Chen, Aling Tang, Shengtao Yan, Guoqiang Zhang

**Affiliations:** 1 Department of Emergency, China-Japan Friendship Hospital, 2 Ying Hua Dong Jie, Chaoyang District, Beijing, 10029 China; 2 Institute of Clinical Medical Sciences, Chinese Academy of Medical Sciences & Peking Union Medical Collage, China-Japan Friendship Hospital, 2 Ying Hua Dong Jie, Chaoyang District, Beijing, 10029 China; 3 Graduate School of Beijing University of Chinese Medicine, No. 11, Bei San Huan Dong Lu, Chaoyang District, Beijing, 10029 China

**Keywords:** Biomarker, Bioinformation, Machine learning, Neuroprotection, Cell death

## Abstract

Neurological prognostication after cardiac arrest (CA) is important to avoid pursuing futile treatments for poor outcome and inappropriate withdrawal of life-sustaining treatment for good outcome. To predict neurological outcome after CA through biomarkers in peripheral blood mononuclear cells, four datasets were downloaded from the Gene Expression Omnibus database. GSE29546 and GSE74198 were used as training datasets, while GSE92696 and GSE34643 were used as verification datasets. The intersection of differentially expressed genes and hub genes from multiscale embedded gene co-expression network analysis (MEGENA) was utilized in the machine learning screening. Key genes were identified using support vector machine recursive feature elimination (SVM-RFE), least absolute shrinkage and selection operator (LASSO) logistic regression, and random forests (RF). The results were validated using receiver operating characteristic curve analysis. An mRNA-miRNA network was constructed. The distribution of immune cells was evaluated using cell-type identification by estimating relative subsets of RNA transcripts (CIBERSORT). Five biomarkers were identified as predictors for neurological outcome after CA, with an area under the curve (AUC) greater than 0.7: CASP8 and FADD-like apoptosis regulator (CFLAR), human protein kinase X (PRKX), miR-483-5p, let-7a-5p, and let-7c-5p. Interestingly, the combination of CFLAR minus PRKX showed an even higher AUC of 0.814. The mRNA-miRNA network consisted of 30 nodes and 76 edges. Statistical differences were found in immune cell distribution, including neutrophils, NK cells active, NK cells resting, T cells CD4 memory activated, T cells CD4 memory resting, T cells CD8, B cells memory, and mast cells resting between individuals with good and poor neurological outcome after CA. In conclusion, our study identified novel predictors for neurological outcome after CA. Further clinical and laboratory studies are needed to validate our findings.

**Supplementary Information:**

The online version contains supplementary material available at 10.1007/s10142-023-01016-0.

## Introduction

Cardiac arrest (CA) is a unique medical emergency characterized by the loss of functional cardiac mechanical activity, which leads to cessation of circulation to the brain tissue. As the brain tissue is highly dependent on consistent oxygen and energy supply, it only takes a few seconds for blood interruption to cause brain tissue injury (Sandroni et al. 2021). Failure to receive treatments will result in death. Under the early initiation of high-quality cardiopulmonary resuscitation and defibrillation, a few patients could return of spontaneous circulation and consciousness. But the rate is very low, only 5.6% in a Danish study (Sondergaard et al. 2020). Most patients remain comatose upon arrival at the hospital and are discharged into intensive care until (Perkins et al. 2021). Despite managements on body temperature, oxygenation, arterial blood pressure, and ventilation, there is still no direct treatment for brain injury. (Cronberg et al. 2020).

For patients who do not wake up promptly, neurological prognostication should be performed no earlier than 72 h after admission to intensive care, to avoid pursuing futile treatments for poor outcome and inappropriate withdrawal of life-sustaining treatment for good outcome. The Cerebral Performance Category (CPC) scale is used to evaluate neurological outcome. This scale consists of 5 categories: (1) good cerebral performance, (2) moderate cerebral performance, (3) severe cerebral performance, (4) coma/vegetative state, (5) brain death (Brain Resuscitation Clinical Trial I Study Group 1986). CPC 1–2 defines a good neurological outcome, while CPC 3–5 indicates a poor neurological outcome (Geocadin et al. 2019). Researchers try to use various methods, such as clinical examination, neuroimaging, electrophysiology, and blood biomarkers to predict neurological outcome. Evidence confirms that the bilateral absence of corneal reflexes, pupillary reflexes, and N20 wave of somatosensory evoked potentials are reliable indicators of poor prognosis (Sandroni et al. 2020). On the other hand, normal blood neuron-specific enolase values, Glasgow Coma Score 4 or 5, N20 wave or continue electroencephalography background, and absent diffusion restriction in the cortex or deep grey matter are indicators of good prognosis after CA (Sandroni et al. 2022). However, blood biomarkers providing quantitative results were fewer, and more predictors in blood are needed.

In recent years, studies on high-throughput functional genomics grow rapidly, providing a powerful tool for discovery disease genes and drug targets. High-throughput genomics data from peripheral blood mononuclear cells (PBMCs) of patients after CA have been published (Stammet et al. 2012; Stefanizzi et al. 2020). But due to the limitation of computing methods, few predictors were found. Multiscale embedded gene co-expression network analysis (MEGENA) is a novel co-expression network analysis framework that can construct large-scale co-expression plane filtering networks and preserve gene interactions to reveal new targets. It has been used in breast carcinoma, lung adenocarcinoma, and tumor cell proliferation (Song and Zhang 2015; Yin et al. 2022). Machine learning algorithms including support vector machine recursive feature elimination (SVM-RFE), least absolute shrinkage and selection operator (LASSO) logistic regression, and random forests (RF) have been used to find key genes in stroke (Zheng et al. 2022) and breast cancer (Yuan et al. 2022). However, few studies have combined MEGENA with machine learning algorithms to find key genes in CA.

In this study, datasets comparing the neurological outcome after CA were downloaded from the Gene Expression Omnibus (GEO). MEGENA, SVM-RFE, LASSO, and RF algorithms were applied to identify potential key biomarkers that could to predict the neurological outcome after CA.

## Methods

### Cardiac arrest dataset

**Fig. 1:**
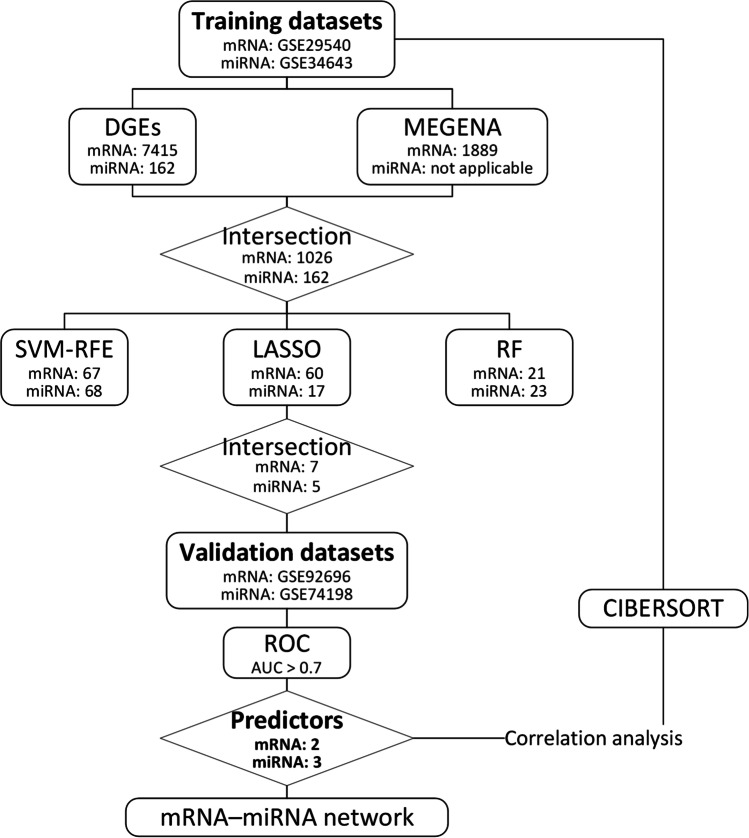
The workflow of our study. AUC, area under the curve; CIBERSORT, cell-type identification by estimating relative subsets of RNA transcripts; DEGs, differentially expressed genes; LASSO, least absolute shrinkage and selection operator; MEGENA, multiscale embedded gene co-expression network analysis; RF, random forests; ROC, receiver operating characteristic; SVM-RFE, support vector machine recursive feature elimination

Four datasets downloaded from the GEO database were used in our study. GSE29546 includes 140 gene expression profiles of blood cells determined using 25,000 ~ gene microarray in two groups of patients: good outcome (CPC 1–2, *n* = 84) and poor outcome (CPC 3–5, *n* = 56). GSE92696 includes 22 participants were dichotomized into good neurological outcome, CPC 1 (*n* = 10), and poor neurological outcome CPC 4 (*n* = 4) and CPC 5 (*n* = 8) were analyzed on whole genome expression microarray to profile post-CA with its innate characteristic molecular signature. GSE74198 includes plasma miRNA profiles of 50 cardiac arrest patients, including 25 good neurological outcome patients (CPC 1) and 25 poor neurological outcome patients (CPC 5) at 6 months. GSE34643 includes plasma miRNA profiles of 10 age- and sex-matched patients, 5 good neurological outcome patients (CPC 1–2) and 5 poor neurological outcome patients (CPC 3–5) at 6 months. GSE29546 and GSE74198 were used as training datasets. GSE92696 and GSE34643 were used as verification datasets. The workflow of our study is shown in Fig. 1.

### Differential expression analysis

The series matrix file of each dataset was downloaded using the GEOquery package (Davis and Meltzer 2007). According to the annotation information in platform, probes were converted to gene symbols or miRNA names. Probes matching multiple genes were deleted. The average value of a gene measured by multiple probes was calculated as the final value. Missing values were filled using multiple imputation method in the mice package (van Buuren and Groothuis-Oudshoorn 2011). The limma (Ritchie et al. 2015) and DESeq2 (Love et al. 2014) packages were used to analyze the differentially expressed mRNA and miRNA, respectively. Differentially expressed genes (DEGs) were identified as adjusted *P* value less than 0.05.

### MEGENA

MEGENA package (Song and Zhang 2015) was used to analyze the co-expression network, dissection into multi-scale functional modules, and network key drivers. We constructed the fast planar filtered network, performed multiscale clustering analysis and multiscale hub analysis, and found the hub genes. Only the mRNA dataset GSE29546 performed MEGENA, due to fewer probes in the miRNA dataset GSE74198.

### Machine learning algorithms

Machine learning algorithms were used to identify key genes in the intersection of DEGs and MEGENA hub genes that can predict good and poor outcomes. The SVM-RFE algorithm was run with the aid of the e1071 package (Meyer et al. 2022) and a script created by Dr. John Colby (https://github.com/johncolby/SVM-RFE). The default parameters were used in this script (cost = 10, cachesize = 500, scale = false, type = “C-classification,” kernel = “linear”). The point with the lowest cross-validation error is the threshold for valuable genes. The LASSO algorithm was run using the glmnet package (Friedman et al. 2010), and a tenfold cross-validation was performed to adjust the optimal penalty parameter. The RF algorithm was run using the randomForest package (Liaw and Wiener 2002). We explored the optimal value of random forest trees and ultimately selected 1000 trees for mRNA and 600 trees for miRNA analysis. The intersection genes of top 30 mean decrease accuracy and top 30 mean decrease gini were considered as valuable genes.

We only included the names of the valuable genes that were used in each machine learning model, regardless of the learning model itself. The intersection genes of valuable genes obtained by three machine learning algorithms were considered as key genes, and the results of three algorithms were visualized by the venn package (Dusa 2022).

### Verification of key genes

The accuracy of each key gene in predicting neurological outcome after CA was validated by analyzing the receiver operating characteristic (ROC) curve and the area under the curve (AUC) using verification datasets; the pROC package (Robin et al. 2011) was used for this analysis. AUC > 0.7 implied a good predictor, and AUC closer to 1 represented better predictive efficiency.

### mRNA-miRNA network

The Pearson correlation coefficient between all genes in GSE29546 and the key mRNA genes was calculated using the expression matrix of GSE29546. Genes with an absolute value of coefficient > 0.6 and *P* < 0.05 were considered as key mRNA-related genes. The Encyclopedia of RNA Interactomes (ENCORI) database (Li et al. 2014) was used to predict the targets of key miRNA genes with the following parameters: CLIP evidence (≥ 5), degradome evidence (≥ 0), program number (≥ 4) and predicted program (none). The mRNA-miRNA network was visualized using Cytoscape 3.8.2 software (Shannon et al. 2003).

### Evaluation of immune cells distribution

We used cell-type identification by estimating relative subsets of RNA transcripts (CIBERSORT) (Newman et al. 2015) to evaluate the difference in the distribution of 22 immune cell types in the blood of patients with good and poor neurological outcome patients after CA by GES29546. The Pearson correlation coefficient of key genes and immune cells was also calculated.

### Statistical analysis

The Wilcoxon rank sum test was used to test the difference between good and poor neurological outcome. Statistical tests were 2-sided, and *P* < 0.05 was considered statistically significant. R 4.2.2 software was used to carry out statistical analyses, and the ggplot2 package (Wickham 2016) was used to visualize the results. The computer hardware used in our study was described in supplementary file 1.

## Results

### CFLAR and PRKX were identified as the key mRNA predictors

**Fig. 2:**
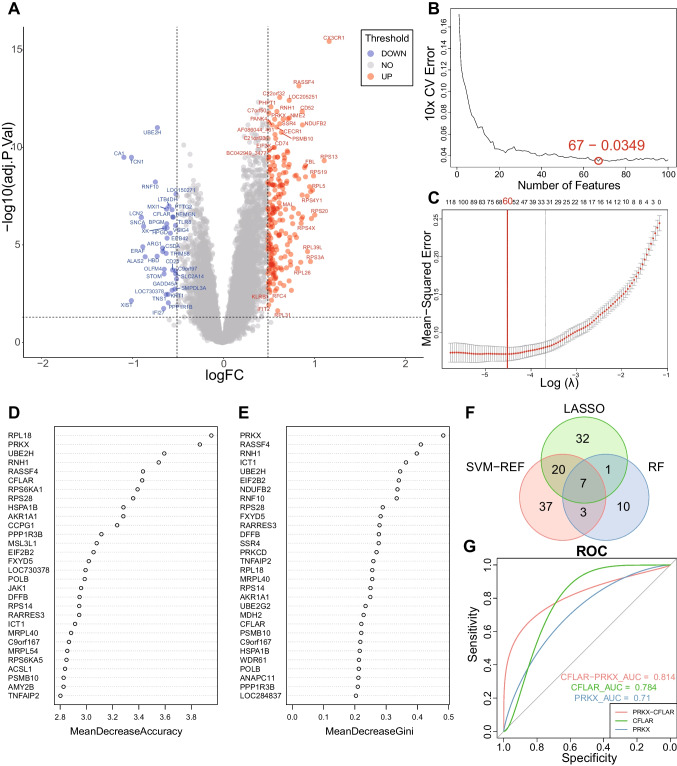
Identification of CFLAR and PRKX as key mRNA predictors. **A** Volcano plot of DEGs in the training dataset. Red represents genes upregulated in the good outcome group, and blue represents genes downregulated in the good outcome group. **B** Error plot of different number of features in SVM-RFE. The minimum error was obtained for the inclusion of 67 genes. **C** Error plot of different lambda in LASSO. The minimum error was obtained for the inclusion of 60 genes. **D**, **E** Top 30 accuracy and gini results in RF respectively. **F** Venn plot of genes identified by the three machine learning algorithms. **G** ROC and AUC of key mRNA predictors in the validation dataset. AUC, area under the curve; DEGs, differentially expressed genes; LASSO, least absolute shrinkage and selection operator; RF, random forests; ROC, receiver operating characteristic; SVM-RFE, support vector machine recursive feature elimination

From the mRNA dataset, we identified 7415 DEGs (Fig. 2A) and 1889 hub genes analyzed by MEGENA. The intersection of DEGs and hub genes resulted in 1026 genes, which were then fed into the three machine learning algorithms. The SVM-RFE algorithm identified 67 genes (Fig. 2B). The LASSO algorithm identified 60 genes (Fig. 2C). The RF algorithm identified algorithm 21 genes (Fig. 2D, E). Of these, there were 7 genes that were identified by all three algorithms (Fig. 2F).

In the validation dataset, the ROC cure analysis showed that the AUC for CASP8 and FADD-like apoptosis regulator (CFLAR) and human protein kinase X (PRKX) was greater than 0.7. For the remaining 5 genes, the AUC was less than 0.7. Interestingly, CFLAR was upregulated in poor neurology outcome group, and PRKX was downregulated, resulting in an AUC of 0.814 when CFLAR minus PRKX (Fig. 2G). The detailed calculation results of each step are shown in supplementary file 2.

### miR-483-5p, let-7a-5p, and let-7c-5p were identified as the key miRNA predictors

**Fig. 3:**
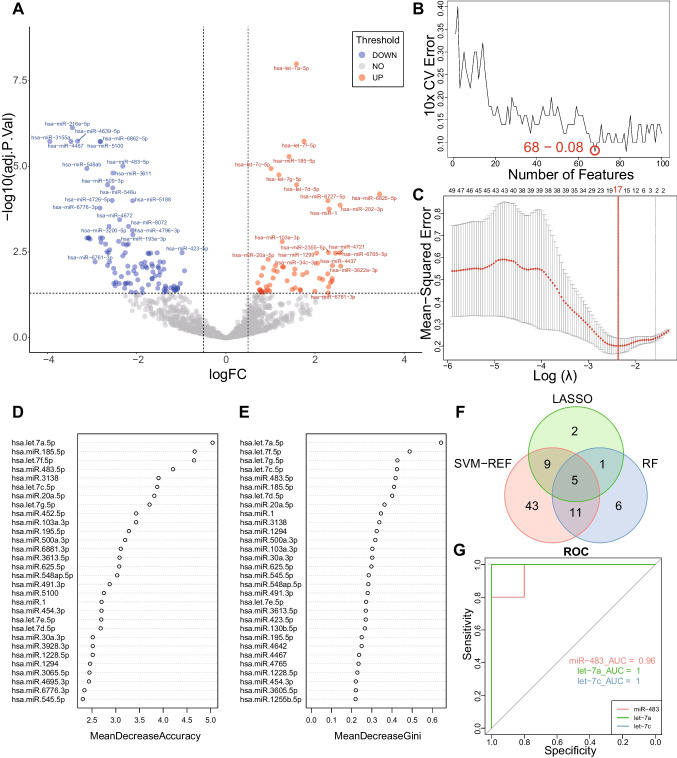
Identification of miR-483, let-7a, and let-7c as key miRNA predictors. **A** Volcano plot of DEGs in the training dataset. Red represents genes upregulated in the good outcome group, and blue represents genes downregulated in the good outcome group. **B** Error plot of different number of features in SVM-RFE. The minimum error was obtained for the inclusion of 68 genes. **C** Error plot of different lambda in LASSO. The minimum error was obtained for the inclusion of 17 genes. **D**, **E** Top 30 accuracy and gini results in RF respectively. **F** Venn plot of genes in the three machine learning algorithms. **G** ROC and AUC of key miRNA predictors in the validation dataset. DEGs, differentially expressed genes; LASSO, least absolute shrinkage and selection operator; PRKX, human protein kinase X; RF, random forests; ROC, receiver operating characteristic; SVM-RFE, support vector machine recursive feature elimination

From the miRNA dataset, we identified 162 DEGs (Fig. 3A) and fed them into the three machine learning algorithms. The SVM-RFE algorithm identified 68 genes (Fig. 3B). The LASSO algorithm identified 17 genes (Fig. 3C). The RF algorithm identified 23 genes (Fig. 3D, E). Of these, there were 5 genes that were identified by all three algorithms (Fig. 3F).

In the validation dataset, the ROC curve analysis showed that the AUC for miR-483-5p, let-7a-5p, and let-7c-5p was greater than 0.7. For the remaining 2 genes, the AUC was less than 0.7 (Fig. 3G). The detailed calculation results of each step are shown in supplementary file 3.

### The mRNA-miRNA network

**Fig. 4:**
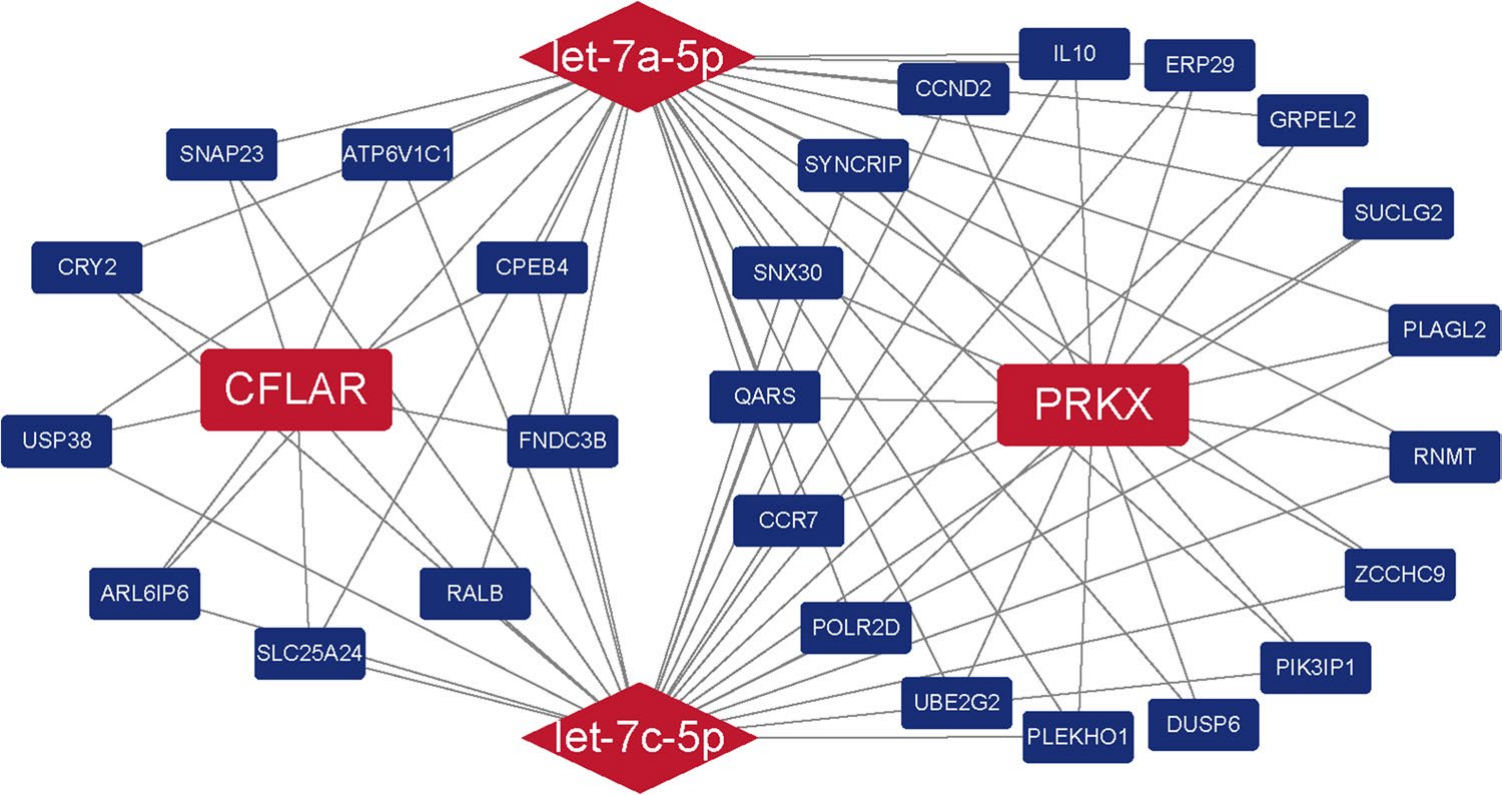
The mRNA-miRNA network. Round rectangles represent mRNA. Diamonds represent miRNA. Red represents the key genes. Blue represents genes related to key genes

The Pearson correlation analysis revealed 246 genes related to CFLAR and 739 genes related to PRKX. In the ENCORI database, miR-483-5p had 2 targets, let-7a-5p had 502 targets, and let-7c-5p had 503 targets. Let-7a-5p and let-7c-5p had similar targets. Unfortunately, there were no predicted targets of key miRNAs on the key mRNA genes. The mRNA-miRNA network had 30 nodes and 76 edges. miR-483-5p had no overlap with CFLAR and PRKX and therefore did not appear in this network. Let-7a-5p had 9 overlapping genes with CFLAR and 17 overlapping genes with PRKX, while let-7c-5p had 9 overlapping genes with CFLAR and 15 overlapping genes with PRKX. Notably, there were no overlapping genes between CFLAR and PRKX (Fig. 4).

### The distribution of immune cells varies with good and poor outcomes: CFLAR and PRKX were related to different immune cells

**Fig. 5:**
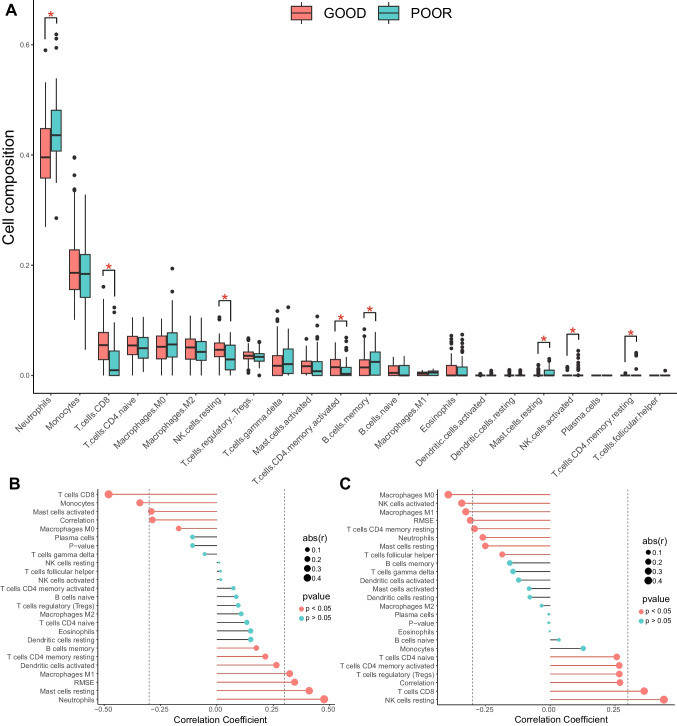
Immune cells in good and poor neurological outcome after CA. **A** Bar plot of different immune cells in good and poor groups. **B** Lollipop chart of correlation between CFLAR and immune cells. **C** Lollipop chart of correlation between PRKX and immune cells

Neutrophils and monocytes were the most common types of immune cells in the blood of patients with CA. There were statistically significant differences in the distribution of neutrophils, NK cells active, NK cells resting, T cells CD4 memory activated, T cells CD4 memory resting, T cells CD8, B cells memory, and mast cells resting between patients with good and poor neurological outcome after CA (Fig. 5A).

CFLAR was related to several immune cell types, including T cells CD8, monocytes, macrophages M1, RMSE, mast cell resting, and neutrophils (Fig. 5B). PRKX was related to macrophages M0, NK cell activated, NK cells resting, macrophages M1, RMSE, and T cells CD8 (Fi. 5C).

## Discussion

In this study, we identified two mRNA genes (CFLAR and PRKX) and three miRNA genes (miR-483, let-7a, and let-7c) as novel predictors for neurological outcome after CA in blood, through the combination of differential expression analysis, MEGENA, and machine learning algorithms. There were few studies using such a comprehensive approach to finding biomarkers in CA.

Previous studies using the same datasets have found several predictors. Stamment and his colleagues found that miR-21 and miR-122 were overexpressed in poor neurological outcome by GSE34643 (Stammetet al. 2012). Eun and his colleagues found that MAPK3, BCL2, and AKT1 were predictors of poor neurological outcome, with an AUC greater than 0.7 by GSE92696 (Eun et al. 2017). Stefanizzi and his colleagues found that miR-9-3p, miR-124-3p, and miR-129-5p could predict neurological outcome by GSE74198 (Stefanizzi et al. 2020). Zhang and his colleagues found that EEF1B2, PSMD14, RBX1, RPFDN5, and SNRPD2 were downregulated and positively correlated with the neurological function of rats by GSE29540 and GSE92696 (Zhang et al. 2022c).

In our study, we used four datasets available in the GEO database on the neurological outcome after CA and used a combination of bioinformatics analysis and machine learning. This could explain why we found different predictors than previous studies. The number of studies using multiple algorithms to identify key genes or biomarkers is growing, and we believe that this will become a new trend (Meng et al. 2022; Zhang et al. 2022a). Actually, it remains challenging to identify predictors for disease outcomes in blood, and further laboratory and clinical researches are needed to validate our results.

CFLAR, also known as cellular caspase 8 (FLICE)-like inhibitory protein (c-FLIP), is widely expressed in the human body. CFLAR can interact with the FADD and caspase 8 to protect cells from apoptosis (Budd et al. 2006; Irmler et al. 1997). What is more, loss of CFLAR results in increased necroptosis and autophagy, suggesting that CFLAR plays an important role in autophagy and necroptosis (He and He 2013). In mice with middle cerebral artery occlusion, CFLAR was decreased. The infract volume in mice increased when CFLAR was knocked out, but decreased significantly when CFLAR was overexpressed. This means that CFLAR may be a potential target for neuroprotection (Xiaohong et al. 2019). Unfortunately, there are few studies on CFLAR and CA. Apoptosis is one of the important pathologies after CA (Zhang et al. 2022b), so we believe that CFLAR, as an apoptosis regulator, has the potential to improve the neurological outcome after CA.

PRKX is a serine/threonine protein kinase regulated by and mediating cAMP signaling in cells, is widely expressed in the human body, and plays an important role in the development of kidney, brain, blood vessels, and blood cells (Huang et al. 2016). It inhibited the activation of Wnt/β-catenin signaling pathway and inhibited ovarian cancer cell malignant, invasion, proliferation, and tumor growth (Chen et al. 2022). PRKX was overexpressed in triple-negative breast cancer (Santuario-Facio et al. 2017) and downregulated in coronary artery disease (Long et al. 2018) which could be a predictor in these diseases. There are few studies on PRKX and CA; more studies are needed to explore this relationship.

MiR-483-5p plays an important role in cancer. MiR-483-5p binds to the fetal mRNA of insulin-like growth factor 2 and enhances its transcription, resulting in tumorigenesis (Liu et al. 2013). Low expression of miR-483-5p was significantly associated with better tongue squamous cell carcinoma patients’ prognosis and neoadjuvant chemosensitivity (Tian et al. 2019). MiR-483-5p can be activated by the Wnt/β-catenin signaling pathway and promotes invasive and metastatic properties of lung adenocarcinoma (Song et al. 2014). MiR-483-5p was overexpressed in metastatic tissues and serum of metastatic patients and could be used as a biomarker for the presence of metastasis (Castro-Vega et al. 2020). But miR-483-5p is a suppressor of liver colonization and metastasis (Loo et al. 2015). Targeting miR-483-5p could prevent the onset of osteoarthritis and delay its progression (Wang et al. 2017). PRKX inhibits β-catenin, but β-catenin activates miR-483-5p, which may be a potential pathway in CA. More studies are needed to confirm this hypothesis.

Let-7a-5p and let-7c-5p are members of the let-7 family of miRNAs. Let-7 is one of the first miRNAs discovered and is well conserved in different animal species. Higher animals have diverse let-7 family members such as let-7a, let-7b, and let-7c (Lee et al. 2016). Previous studies show that let-7 is a tumor suppressor and has crosstalk with oncogenes including β-catenin, which can inhibit let-7. Exogenous expression of let-7 inhibits the apoptosis mediated by Fas, implying an anti-apoptotic effect (Wang et al. 2015). More studies are needed to uncover the content of let-7 and CA.

However, our study has several limitations. The datasets of CA used in our study are mainly microarray-based data, which is limited by the pre-designed markers and a limited number of markers (Povysil et al. 2019). There are many missing values in the microarray-based datasets, which may lead to a decline in the credibility of our results. Nowadays, with the advancement of next-generation sequencing, the cost is dropping. New technologies such as single-cell RNA-sequencing help to understand the pathology of diseases and cell-to-cell interactions (Su et al. 2022). which can be used to understand CA and find biomarkers and targets. What is more, our results were only validated by other datasets and not in cell lines or animal models. In further studies, in vitro and in vivo studies using CA models are needed to confirm our findings. Clinical trials enrolling CA patients are needed to confirm the predictive value of these key genes.

In conclusion, our study identified CFLAR and PRKX as novel mRNA predictors and miR-483-5p, let-7a-5p, and let-7c-5p as novel miRNA predictors for neurological outcome after CA. The distribution of immune cells varies with different outcomes. CFLAR and PREKX were related to different immune cells. More clinical and laboratory studies are needed to confirm our findings.

## Supplementary Information

Below is the link to the electronic supplementary material.

Supplementary file1 (TXT 0 KB)

Supplementary file2 (XLSX 2426 KB)

Supplementary file3 (XLSX 86 KB)

## Data Availability

Data will be shared with qualified investigators upon request, please contact corresponding authors. The code used in our study is available at https://gitee.com/lzh23/GEO1.

## Declarations

### Disclaimer

The funder played no role in our study.

## Abbreviations

AUC: Area under the curve
CA: Cardiac arrest
CFLAR: CASP8 and FADD-like apoptosis regulator
CIBERSORT: Cell-type identification by estimating relative subsets of RNA transcripts
CPC: Cerebral performance category
DEGs: Differentially expressed genes
ENCORI: Encyclopedia of RNA interactomes
GEO: Gene expression omnibus
LASSO: Least absolute shrinkage and selection operator
MEGENA: Multiscale embedded gene co-expression network analysis
PRKX: Human protein kinase X
RF: Random forests
ROC: Receiver operating characteristic
SVM-RFE: Support vector machine recursive feature elimination
