# Correction to: Identification of predictors for neurological outcome after cardiac arrest in peripheral blood mononuclear cells through integrated bioinformatics analysis and machine learning

**DOI:** 10.1007/s10142-024-01440-w

**Published:** 2024-09-25

**Authors:** Zhonghao Li, Ying Qin, Xiaoyu Liu, Jie Chen, Aling Tang, Shengtao Yan, Guoqiang Zhang

**Affiliations:** 1 Department of Emergency, China-Japan Friendship Hospital, 2 Ying Hua Dong Jie, Chaoyang District, Beijing, 10029 China; 2 Institute of Clinical Medical Sciences, Chinese Academy of Medical Sciences & Peking Union Medical Collage, China-Japan Friendship Hospital, 2 Ying Hua Dong Jie, Chaoyang District, Beijing, 10029 China; 3 Graduate School of Beijing University of Chinese Medicine, No. 11, Bei San Huan Dong Lu, Chaoyang District, Beijing, 10029 China

**Correction to: Functional & Integrative Genomics (2023) 23:83**

10.1007/s10142-023-01016-0

Originally, the paper was published with error. The GSE29546 mentioned in the article is incorrect and should be corrected to GSE29540. Figure 1 also be corrected accordingly.

**Fig. 1:**
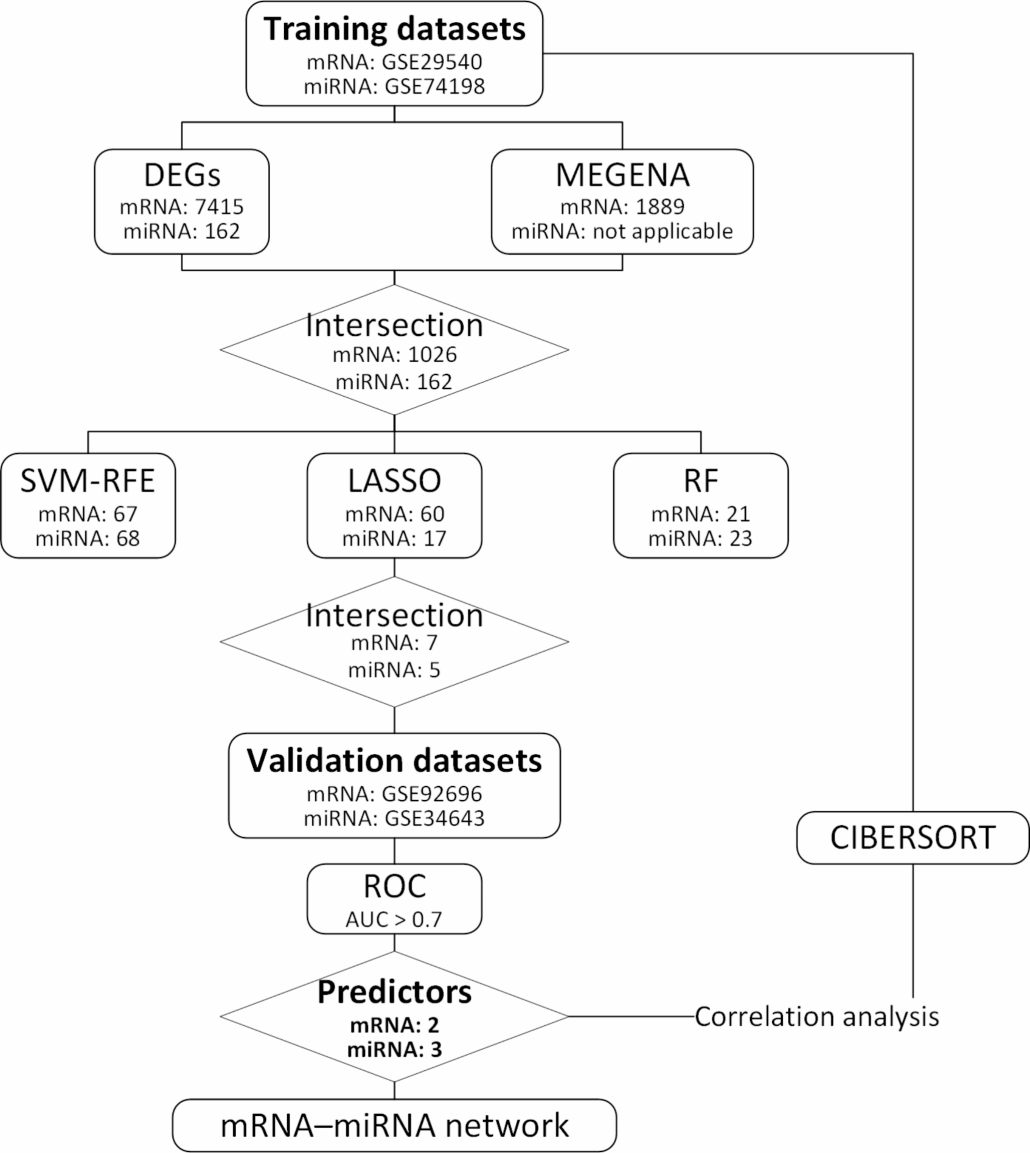
The workflow of our study. AUC, area under the curve; CIBERSORT, cell-type identification by estimating relative subsets of RNA transcripts; DEGs, differentially expressed genes; LASSO, least absolute shrinkage and selection operator; MEGENA, multiscale embedded gene co-expression network analysis; RF, random forests; ROC, receiver operating characteristic; SVM-RFE, support vector machine recursive feature elimination

